# The preoperative geriatric nutritional risk index (GNRI) is an independent prognostic factor in elderly patients underwent curative resection for colorectal cancer

**DOI:** 10.1038/s41598-022-07540-6

**Published:** 2022-03-07

**Authors:** Tamuro Hayama, Yojiro Hashiguchi, Tsuyoshi Ozawa, Makoto Watanabe, Yoshihisa Fukushima, Ryu Shimada, Keijiro Nozawa, Keiji Matsuda, Shoichi Fujii, Takeo Fukagawa

**Affiliations:** 1 Department of Surgery, Teikyo University School of Medicine, 2-11-1 Kaga, Itabashi-ku, Tokyo, 173-8605 Japan; 2 Department of Surgery, Yokohama General Hospital, Yokohama, Japan

**Keywords:** Gastrointestinal cancer, Tumour biomarkers

## Abstract

The world is becoming longer-lived, and the number of elderly colorectal cancer patients is increasing. It is very important to identify simple and inexpensive postoperative predictors in elderly colorectal cancer patients. The geriatric nutritional risk index (GNRI) is a marker of systemic nutrition and is associated with poor survival in various kinds of cancers. A few reports have investigated recurrence factors using preoperative GNRI with CRC (colorectal cancer) patients. This study aimed to investigate whether preoperative GNRI is associated with recurrence-free survival (RFS) and overall survival (OS) in elderly patients with CRC. This study retrospectively enrolled 259 patients with Stage I–III CRC who were more than 65 years old and underwent curative surgery at a single institution in 2012–2017. We classified them into low GNRI (RFS: ≤ 90.5, OS ≤ 101.1) group and high GNRI (RFS: > 90.5, OS > 101.1) group. Multivariable analyses showed low GNRI group was an independent risk factor for 3-year RFS (*P* = 0.006) and OS (*P* = 0.001) in the patients with CRC. Kaplan–Meier analysis showed 3-year RFS and 3-year OS were significantly worse in the low GNRI group than in high GNRI group (*p* = 0.001, 0.0037). A low-preoperative GNRI was significantly associated with a poor prognosis in elderly CRC patients.

## Introduction

Life expectancy in humans is increasing. Expansion of the worldwide population and elevation of life expectancy have increased the number of elderly individuals^1^.

When asked if an elderly CRC (colorectal cancer) patient wants treatment, most patients do. We know that increasing age also increases the risk for complications during and after colorectal surgery. Aging itself can reduce physiological recuperative power, aging is an independent risk factor for both in-hospital morbidity and mortality after colorectal surgery^2,3^. Recently, it has been widely accepted that GNRI (Geriatric Nutritional Risk Index) is strongly associated with mortality in elderly patients with various cancers^4–6^. However, as far as we know, there are few reports on the prognostic significance of GNRI in patients with colorectal cancer. This study investigated whether GNRI is a useful predictor of recurrence and long-term survival in elderly patients with colorectal cancer who have undergone curative resection.

## Results

### Patient characteristics

Our study included a total of 259 patients. The median age was 74.2 (range 65–93) years; 144 (55.6%) patients were male and 115 (44.4%) were female. T factor (the depth of tumor invasion) was 77 (29.7%) for T1 or T2, and 182 (70.3%) for T3 or T4. There were 87 (33.6%) cases with lymph node metastasis (N factor +) and 172 (66.4%) cases without lymph node metastasis (N factor −). There were 89 (34.5%) cases with high preoperative CEA levels and 41 (15.9%) cases with high preoperative CA19-9 levels. The low GNRI group were 50 (19.5%) patients, high GNRI group were 209 (80.5%) (Table.1).

**Table 1:** Clinicopathological features of the stage I–III colorectal cancer patients who underwent curative tumor resection.

Variables	n = 259 (%)
Age, years (≤ 74, > 74)	143 (55.2)/116 (44.8)
Gender (male/female)	144 (55.6)/115 (44.4)
BMI (≤ 22/ > 22)	124 (47.9)/135 (52.1)
Tumor location (right side/left side)	99 (38.2)/160 (61.8)
Histology (well or moderate/others)	228 (88.0)/31 (12.0)
Depth of tumor invasion (T1·T2/T3·T4)	77 (29.7)/182 (70.3)
Lymph node metastasis (+ / −)	87 (33.6)/172 (66.4)
Lymph invasion (+ / −)	113 (43.6)/146 (56.3)
Venous invasion (+ / −)	179 (69.1)/80 (30.9
CEA level (high/normal)	89 (34.5)/169 (65.5)
CA19-9 level (high/normal)	41 (15.9)/218 (84.1)
GNRI (low/high)	50 (19.5)/209 (80.5)

### GNRI cut-off value

**Figure 1:**
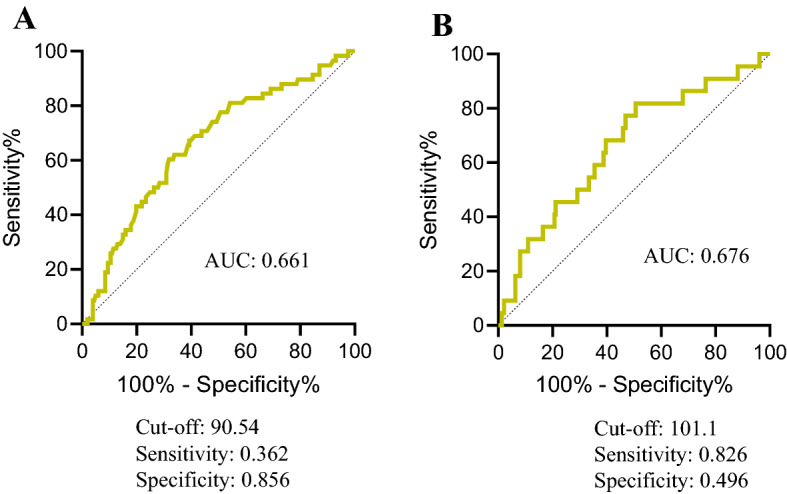
ROC for GNRI as a predictive factor for postoperative survival was plotted to verify the optimum cutoff value of GNRI. (**A**) Relapse-free survival, (**B**) overall survival.

We performed ROC analyses to define the optimal cut-off value of the preoperative GNRI. ROC analyses for the 3-year RFS showed that 90.5 was the cut-off value of the GNRI that could discriminate CRC patients with recurrence from those without recurrence from those, with an area under the curve (AUC) of 0.62 (sensitivity: 0.36, specificity: 0.86 (Fig. 1A). ROC analyses for the 3-year OS showed that 101.1 was the cut-off value of the GNRI that could discriminate CRC patients with a poor prognosis from those without a poor prognosis from those with AUC of 0.68 (sensitivity: 0.83, specificity: 0.50 (Fig. 1B).

### Associations of GNRI quality with clinicopathological factors

Correlation between GNRI and various clinicopathological factors includes gender, age, BMI, pT stage, pN stage, lymph/venous invasion, tumor location, pathological type, CEA levels and CA19-9 levels are included. GNRI was significantly correlated with pT stage (*p* < 0.0001), BMI (*p* < 0.0001), tumor location (right side: cecum, ascending colon, transvers colon, left side: descending colon, sigmoid colon, rectum) (*p* = 0.017) and CA19-9 (*p* < 0.0001) (Table 2).

**Table 2:** The relationship between GNRI status and clinicopathological factors in the elderly colorectal cancer patients.

Variables	GNRI low group (n = 51)	GNRI high group (n = 207)	*p*-value
Age (≤ 74, 74 <)	24 (47.1)/27 (52.9)	119 (57.5)/88 (42.5)	0.191
Gender (male/female)	28 (56.0)/23 (44.0)	116 (56.0)/91 (43.4)	0.996
BMI (≤ 22/22 <)	41 (80.4)/10 (19.6)	84 (40.6)/123 (59.4)	0.0001
Tumour location (Right side/Left side)	27 (52.9)/24 (47.1)	72 (34.6)/135 (65.4)	0.017
Tumour location (colon/rectum)	5 (9.8)/46 (90.2)	41 (19.7)/166 (80.3)	0.100
Histology (Well or Moderate/Others)	10 (19.6)/41 (80.4)	21 (10.1)/186 (89.9)	0.076
Depth of tumor invasion (T1·T2/T3·T4)	48 (96.0)/3 (4.0)	133 (64.6)/74 (35.4)	0.0001
Lymph node metastasis (+ / −)	19 (38.0)/32 (62.0)	67 (32.4)/140 (67.6)	0.452
Lymph invasion (+ / −)	27 (52.0)/24 (48.0)	86 (41.6)/121 (58.5)	0.182
Venous invasion (+ / −)	38 (74.0)/13 (26.0)	140 (67.6)/67 (32.4)	0.377
CEA lebel (high/normal)	18 (36.0)/33 (64.0)	71 (34.5)/136 (65.5)	0.838
CA19-9 lebel (high/normal)	18 (36.0)/33 (64.0)	22 (10.7)/185 (89.3)	0.0001

### ALB and %IBW (ideal body weight) scattergraphs by GNRI

For albumin, the preoperative blood albumin concentration is used. % IBW is calculated by current weight/ideal weight (22 × height (m)^2^) × 100. The mean values of the low GNRI group were as follows. ALB: 4.0, %IBW: 106.2, and those of the high GNRI group were 3.1, 89.8. All items were significantly different between the two groups.

### Survival analysis of GNRI in elderly CRC patients

The total of 259 patients with a median follow-up of 1214 days (interquartile range 7–2490 days) developed disease recurrence 43 (19.4%). Among the 43 patients with recurrence, liver metastases were observed in 14 (32.6%), lung metastases in 12 (27.9%), peritoneal carcinomatosis in 6 (14.0%), local recurrence in 4 (9.3%), para-aortic lymph nodes in 4 (9.3%), others in 6 (14.0%).

### Univariate and multivariate analysis of predictive factors for 3-year RFS

All patients were categorized into the low GNRI group (< 90.54; n = 51, 19.8%) or high GNRI group ((≥ 90.54; n = 208, 80.2%). We examined GNRI and clinicopathological factors in 3-year RFS. The GNRI, histological grade, lymph invasion, vascular invasion, pT category, pN category, preoperative CEA level, and CA19-9 level were significantly associated with poor RFS in the univariate survival analyses (Table 3). Other factors including age, gender, tumor location, BMI were not significantly associated with 3-year RFS. The multivariate analysis identified GNRI, pT category, pN category, preoperative CEA level as independent prognostic factors associated with 3-year RFS (Table 3).

**Table 3:** The univariate and multivariate analysis of prognostic factors for 3-year RFS.

Variables	Univariate			Multivariate		
	HR	95%CI	*p*-value	HR	95%CI	*p*-value
Age (≤ 74, 74 <)	1.23	0.67–1.58	0.721			
Gender (male/female)	1.18	0.50–1.42	0.523			
BMI (≤ 22/22 <)	1.48	0.89–2.50	0.131			
Tumour location (Right side/Left side)	0.91	0.54–1.55	0.749			
Histology (Well or Moderate/Others)	1.9	1.26–4.51	0.007	2.43	1.24–4.78	0.010
Depth of tumor invasion (T1·T2/T3·T4)	14.6	3.57–60.0	0.0002	4.98	1.50–16.6	0.009
Lymph node metastasis (− / +)	4.00	2.34–6.82	0.0001	2.78	1.51–5.10	0.001
Lymph invasion (− / +)	3.31	1.90–5.78	0.0001	1.84	0.99–3.42	0.053
Venous invasion (− / +)	2.24	1.16–4.31	0.016	1.28	0.64–2.56	0.490
CEA lebel (normal/high)	2.18	1.300–3.64	0.004	2.13	1.19–3.82	0.011
CA19-9 lebel (normal/high)	3.04	1.74–5.32	0.0003	1.09	0.58–2.04	0.80
GNRI (low/high)	2.87	1.68–4.91	0.0003	2.31	1.28–4.20	0.006

### Univariate and multivariate analysis of predictive factors for 3-year OS.

We set 101.1 and cut off values using the ROC curve and youden index. The control group was divided into two groups using the cut off value. All patients were categorized into the low GNRI group (≤ 101.1; n = 138, 53.2%) or high GNRI group (> 101.1; n = 121, 46.8%). The results of the univariate and multivariate analyses for 3-year OS are summarized in Table 4. In the univariate analyses, histological grade, lymph invasion, pT category, pN category, preoperative CEA level, and GNRI were significantly associated with 3-year OS (*p* = 0.004, 0.0007, 0.020, 0.0007, 0.013, 0.005). In multivariate analyses for 3-year OS, histological grade, lymph invasion, preoperative CEA level and GNRI were independent predictive factors (*p* = 0.006, 0.025, 0.009, 0.012). Lymph invasion and lymph node metastasis may be correlated. As a result, lymph node metastasis may not have been significant.

**Table 4:** The univariate and multivariate analysis of prognostic factors for 3-year OS.

Variables	Univariate			Multivariate		
	HR	95%CI	*p*-value	HR	95%CI	*p*-value
Age (≤ 74, 74 <)	1.24	0.55–2.82	0.600			
Gender (male/female)	1.09	0.47–2.53	0.835			
BMI (≤ 22/22 <)	1.49	0.65–3.37	0.353			
Tumour location (Right side/Left side)	1.89	0.83–4.28	0.129			
Histology (Well or Moderate/Others)	3.71	1.53–9.02	0.004	3.68	1.44–9.39	0.006
Depth of tumor invasion (T1·T2/T3·T4)	10.91	1.47–81.0	0.020	4.76	0.63–36.2	0.132
Lymph node metastasis (− / +)	4.67	1.92–11.4	0.0007	2.69	0.99–7.31	0.053
Lymph invasion (− / +)	6.52	2.22–19.2	0.0007	3.85	1.18–12.5	0.025
Venous invasion (− / +)	1.83	0.68–4.93	0.231			
CEA lebel (normal/high)	2.83	1.24–6.46	0.013	3.15	1.33–7.44	0.009
CA19-9 lebel (normal/high)	1.73	0.64–4.67	0.279			
GNRI (low/high)	4.77	1.62–14.03	0.005	4.18	1.37–12.8	0.012

### TNM Stage in low GNRI group and High GNRI group

There were 66 patients (25.4%) with stage I, 109 (42.1%) with stage II, and 84 (32.4%) with stage III cancer. With RFS and OS, the number of patients with low GNRI tended to increase statistically significantly as the stage progressed (RFS: *p* = 0.0003, OS: *p* = 0.0004) (Table 5).

**Table 5:** Correlation between colorectal cancer stage and GNRI status.

	RFS			OS		
	Low GNRI	High GNRI	*P*-value	Low GNRI	High GNRI	*P*-value
Stage I	2 (0.77)	64 (24.7)	0.0003	22 (8.5)	44 (17.0)	0.0004
Stage II	30 (11.6)	79 (30.5)		70 (27.0)	39 (15.1)	
Stage III	19 (7.3)	65 (25.1)		46 (17.8)	38 (14.7)	

### Kaplan–Meier curve of GNRI in elderly

**Figure 2:**
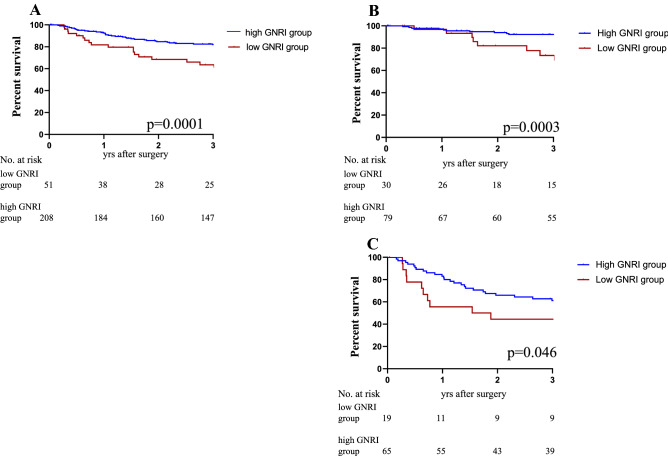
Kaplan–Meier analysis for the RFS of colorectal cancer patients in all stages according to GNRI (**A**) and stratification analysis based on TNM stage: stage I, stage II (**B**) and stage III (**C**).

**Figure 3:**
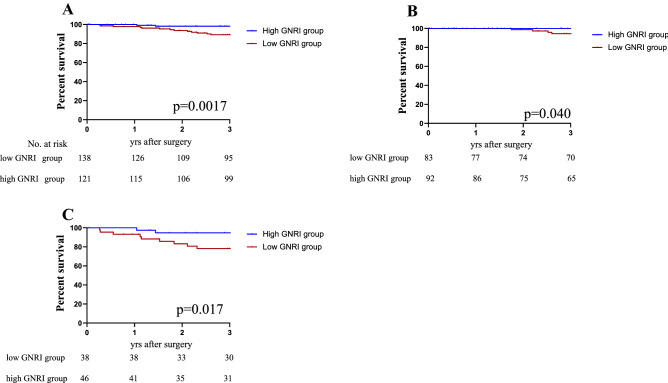
Kaplan–Meier analysis for the OS of colorectal cancer patients in all stages according to GNRI (**A**) and stratification analysis based on TNM stage: stage I, stage II (**B**) and stage III (**C**).

Survival analyses were performed between low GNRI group and high GNRI group according to cutoff value of GNRI. Statistically significant differences between the two groups were revealed by Kaplan–Meier curves on both 3-year RFS (*P* < 0.0001) and 3-year OS (*P* < 0.004), indicating a potential prognostic value of GNRI. The 3-year RFS were 62.1% for the low GNRI group, 82.1% for the high GNRI group, respectively (Fig. 2A). Furthermore, according to the TNM staging stratification analysis, the patients with a low GNRI group were closely associated with poor prognosis stages I + II and III (*P* = 0.0003, *p* = 0.046; Fig. 2B,C). The TNM staging analysis was performed by adding Stage I and Stage II due to the small number of Stage I. The 3-year OS were 85.4% for the low GNRI group, 95.3% for the high GNRI group, respectively (Fig. 3A). In OS, the patients with a low GNRI group were closely associated with poor prognosis stages I + stage II and III (*P* = 0.040, *p* = 0.017; Fig. 3B,C).

### Comparison with other nutritional indicators using ROC curve

**Figure 4:**
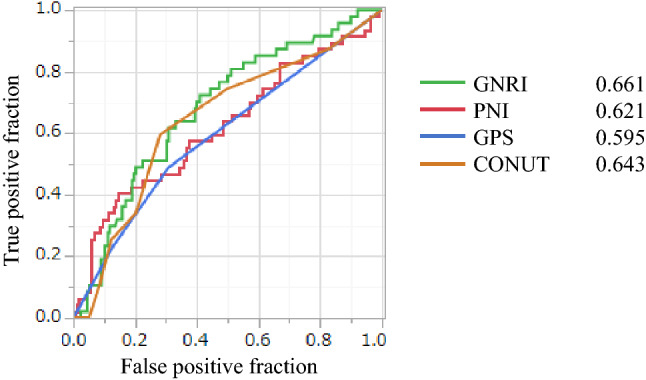
Comparison of ROC curves using nutritional markers.

ROC analysis was performed using PNI, GPS, and CONUT scores, which are nutritional markers that have been reported to be associated with cancer recurrence, and AUC was calculated. As a result, AUC (area under the curve) had the highest GNRI with GNRI of 0.661, PNI of 0.621, GPS of 0.595, and CONUT of 0.643 (Fig. 4), GNRI was the best predictor of RFS in cases with CRC.

## Discussion

Many studies have reported that nutrition-related factors and host immunity have a strong impact on the prognosis of cancer patients^7,8^. The GNRI was firstly reported that simple and accurate tool for predicting the risk of morbidity and mortality in hospitalized elderly patients^9^. The GNRI was strongly associated with mortality in elderly hospitalized patients and in patients with various cancers^10–13^. In our study, a survival analysis of stage I–III CRC patients who underwent curative surgery revealed that patients with low GNRI had significantly worse 3-year RFS than those with high GNRI. Similarly, in the 3-year OS, the prognosis was poor in the low GNRI group. The GNRI was also an independent risk factor for 3-year RFS and 3-year OS in multivariate analysis.

The underlying mechanism by which the low GNRI group results in poor prognosis among colorectal cancer patients undergoing curative surgery is unknown. Two factors can be inferred for the poor prognosis of the low GNRI group. The GNRI is composed of serum albumin levels and body weight (actual body weight [ABW]/IBW) and represents malnutrition.

First, cancer patients are prone to malnutrition, showing a reduced anabolic response to nutritional support. Anabolic resistance refers to the resistance to assimilation in which protein synthesis in muscle tissue does not occur normally after ingesting nutrients such as amino acids due to surgery, trauma, chronic debilitating diseases, aging, etc.^14^. This also occurred in CRC patients, and it has been reported that a blunted reaction of muscle protein synthesis was observed in CRC patients after injection of the amino acid mixture^15^. Second, albumin synthesis may be suppressed in patients with CRC. There are GPS (Glasgow Prognostic Score) and PNI (prognostic nutritional index) in the score of nutritional evaluation using albumin. The GPS is a score using serum albumin level and CRP (C-reactive protein). It has been reported that when the serum albumin level is low, GPS becomes high and the prognosis was poor in postoperative patients with CRC^16^. The PNI is a score calculated using lymphocyte count and serum albumin level. Tominaga et al. reported a poor prognosis for patients with postoperative CRC with low preoperative PNI^17^. Thus, low serum albumin levels have been reported to have a poor prognosis. Hypoalbuminemia induces an impaired immune response, and immunity had influence on cancer prognosis^18^. Additionally, a low serum albumin levels was associated with elevated inflammatory cytokines such as interleukin-1, and interleukin-6, tumor necrosis factor-alpha, CRP which may lead to the progression of CRC^19^. Therefore, a low GNRI may reflect impaired tumor immunity which may cause cancer progression.

The TNM stage has been widely used as the most applicable postoperative staging evaluation system for various cancers worldwide, and it plays an important guiding role in postoperative follow-up and treatment for CRC patients^20,21^. However, it is often reported that there is significant survival heterogeneity among CRC patients with the same TNM stage, and that the TNM stage is inadequate in individual prognosis prediction^22,23^. This may be because the TNM stage only classifies patients according to postoperative pathological results but does not include the patient’s own nutritional status. In recent years, we have focused on the tumor environment from the tumor itself, especially the nutritional and inflammatory status of the patient^7,23,24^. By classifying colorectal cancer patients by stage and using GNRI, the ability to discriminate prognosis was improved. Therefore, we believe that GNRI can effectively complement the TNM stage and play an important role in assessing the individual prognosis of CRC patients.

This study has some limitations. First, this study was retrospective in design and included patients from a single institution. Overcoming potential biases in observational studies requires controlled randomized controlled trials comparing each GNRI risk group. Second, this study has undergone surgery for a variety of colorectal cancers and does not take into account differences between surgical procedures. Third, there is no consensus regarding the GNRI cut-off value, and this makes it difficult to use the GNRI in clinical settings. We selected the GNRI herein by using a ROC analysis. The GNRI is a non-specific marker of nutrition, which implies that another systemic disease can affect the GNRI. Our study findings need further review and validation in more CRC patients.

## Conclusions

Our study provided novel evidence for the clinical relevance and potential feasibility of GNRI as a prognostic biomarker in CRC. Assessment of our developed GNRI could identify patients with elderly CRC who have a poor prognosis.

## Patients and methods

### Patient selection

Stage I–III CRC diagnosed based on the 8th edition of the United States Joint Commission on Cancer (AJCC)^25^ staging system and undergoing curative resection at Teikyo University Hospital from 2012 to 2017. We enrolled 259 patients with Stage I–III CRC aged ≥ 65 years. The surgery of all of the patients was elective. This study has been approved by Teikyo University Ethics (Registration Number; 19-153).

### Nutritional assessment by GNRI (Geriatric Nutritional Risk Index)

The GNRI was calculated that [(14.89 × albumin (mg/dl)] + [41.7 × (present/ideal body) weight (kg)]. The present/ideal body weight value was set to 1 when the patient’s body weight exceeded the ideal body weight^26^. The ideal body weight was defined as a body mass index of 22 kg/m^2^^26^.

### Other nutritional markers (Prognostic Nutritional Index: PNI, GPS, CONUT score)

PNI is a nutritional index proposed by Onodera et al.^27^. And is calculated using serum albumin and total lymphocyte count. PNI = 10 × Alb + 0.005 × total lymphocyte count.

Initially, it was reported as a risk predictor of perioperative complications, later it was reported that evaluation of preoperative PNI was useful as a predictor of prognosis in cancer patients^28^. Glasgow Prognostic Score (GPS) was published by McMillan et al. In 2003. This is a classification using the nutritional index proposed for the first time in non-small cell lung cancer^29^. It was reported to be a better prognostic marker than classification based on stage and performance status. The CONUT score is used as a nutritional evaluation index calculated by scoring albumin level, total lymphocyte count, and total cholesterol level^30^. We have previously reported that CONUT score is useful as a predictor of prognosis after colorectal cancer surgery^25^.

### Survival follow-up

Surgical resection was defined as curative when there was no evidence of tumor recurrence and the distant metastases were histologically and macroscopically complete. Patients were followed up every 3 months for the first 3 years, every 6 months for the next 2 years. At each follow-up, all patients underwent physical examination and measurements of serum CEA (carcinoembryonic antigen) and CA19-9 (carbohydrate antigen 19-9). They also underwent colonoscopy 1–2 years after surgery (rectal cancer was every year after surgery). Thoraco-abdominal computed tomography scans were usually taken every 6 months. Recurrence was defined as the appearance of a radiological, clinical, and/or pathological diagnosis of cancer cells that were local or distant from their original location.

### Determination of cut-off values

The cut-off value for the GNRI was defined according to the receiver-operating characteristic (ROC) curve analysis with Youden’s index for the survival, and for BMI 22, for CEA (5 ng/ml) and CA19-9 (37 U/ml) were the upper limit of the normal range in our institute.

### Statistical analysis

Differences in categorical variables were examined using a chi-square test or Fisher's exact test. Relapse-free survival (RFS) was calculated from the date of the patient underwent surgery to that of recurrence or death, overall survival (OS) was calculated from the date of the patient underwent surgery to that of death, using the Kaplan–Meier method. Univariate and multivariate analyses were performed using a Cox proportional hazards regression model for RFS and OS. Multivariate analyses were performed using the factors that were significant in univariate analyses. Clinical variables that were considered for univariate and multivariate analyses, in addition to the target GNRI, were previously identified confounding factors with an impact on the prognosis with CRC: sex, age at the diagnosis, histology, pathological T stage (T1/2 or T3/4), lymph-node metastasis (present or absent), BMI (≥ 22 or < 22), CEA levels (< 5.0 vs. ≥ 5.0 ng/mL), CA-19–9 levels (< 37 vs. ≥ 37 U/mL). Probability (*p*)-values ≤ 0.05 were considered significant. All statistical analyses were performed using JMP 15 software (SAS, Cary, NC, USA).

### Human and animal rights

All procedures performed in this study involving human participants were in accordance with the 1964 Helsinki declaration and its latest amendments and comparable ethical standards. These authors do not perform a study with animals.

### Ethics approval

This study has been approved by Teikyo University comittee (Registration Number; 19-153).

### Consent to participate

A written informed consent was obtained from all individual participants included in the study.

## Data Availability

All data generated or analysed during this study are included in this article. Further enquiries can be directed to the corresponding author.
